# Potential non-invasive detection of lesions in broiler femur heads: application of the DXA imaging system

**DOI:** 10.3389/fphys.2024.1363992

**Published:** 2024-05-17

**Authors:** Alison Ramser, Elizabeth S. Greene, Robert Wideman, Sami Dridi

**Affiliations:** University of Arkansas, Center of Excellence for Poultry Science, Fayetteville, AR, United States

**Keywords:** imaging, dual-energy X-ray absorptiometry, broiler, femoral head necrosis, non-invasive

## Abstract

Leg health is a significant economic and welfare concern for the poultry industry. Current methods of detection rely on visual assessment of the legs and gait scores and bone scoring during necropsy for full characterization. Additionally, the current scoring of femurs only examines the external surface of the femoral head. Through the use of the dual-energy X-ray absorptiometry (DXA) imaging system, we show the presence of a necrotic region in the femurs that would otherwise be considered healthy based on the current evaluation procedures. Importantly, these lesions were present in almost 60% (22 of 37) of femurs that scored normal for femoral head necrosis (FHN). Additionally, these femurs showed greater bone mineral content (BMC) relative to weight compared to their counterparts with no lucent lesions (6.95% ± 0.20% vs. 6.26% ± 0.25; *p* = 0.038). Identification of these lesions presents both a challenge and an opportunity. These subclinical lesions are likely to be missed in routine scoring procedures for FHN and can inadvertently impact the characterization of the disease and genetic selection programs. Furthermore, this imaging system can be used for *in vivo*, *ex vivo*, and embryonic (egg) studies and, therefore, constitutes a potential non-invasive method for early detection of bone lesions in chickens and other avian species.

## 1 Introduction

Although leg health issues in broilers and other meat-type avian species have been documented for decades, their pervasiveness and consequences have risen in recent years alongside faster growth rates and higher meat yields (McNamee and Smyth, 2000; Granquist et al., 2019). Even with the addition of genomic selection and advances in phenotypic measurements in primary breeder flocks, leg health continues to be a major animal welfare and performance concern (Bradshaw et al., 2002). The reported incidence varies from 15% to 30% incidence in broiler flocks worldwide (Knowles et al., 2008; Kaukonen et al., 2017; Wijesurendra et al., 2017; Granquist et al., 2019). Academia and industry alike have sought to better understand and define leg health, particularly the most common diseases, infections, and injuries that can plague a commercial flock. femoral head or tibial head necrosis (FHN/THN), also known as bacterial chondronecrosis with osteomyelitis (BCO), is one of the leading causes of lameness, in which the integrity of the growth plate and often the adjacent regions is compromised due to bacterial infection, necrosis, and overall bone attrition (Wideman, 2016). It is well-documented that the pathology in a commercial setting is derived from a combination of weak bone and bacterial translocation and infection; however, in experimental conditions, it can be spontaneously induced via a wire-flooring and bacterial challenge model (Wideman et al., 2012; Gilley et al., 2014; Packialakshmi et al., 2015). The presence of FHN is only determined via necropsy, and the subclinical incidence of FHN (the presence of lesions without lameness) has also been documented (Wideman et al., 2012). These facets of FHN pathology have contributed to its persistence in broilers, as geneticists rely on sibling data and have also calculated low heritability based on the current means of phenotyping FHN (Siegel et al., 2019). Therefore, improving the means of measuring and characterizing FHN is vital for refining genetic selection, targeting preventative measures, and assessing potential treatments.

As technology for *in vivo* imaging has become faster, more accurate, and more affordable, it has become a more viable option for monitoring and characterizing disease states and phenotypes. Research on the use of dual-energy X-ray absorptiometry (DXA) imaging (Hester et al., 2004), X-ray imaging (Zheng et al., 2023), computerized tomography (CT) scanning (Aguado et al., 2015), and ultrasound (Mutalib et al., 1996) in poultry has been reported and is being expanded upon almost yearly. In the present study, we utilized a small, self-shielding DXA and X-ray machine to visualize and characterize the leg quarters of broilers. It was through these images that evidence of regions of bone attrition and potential necrosis was first seen as areas with hyposignal density in the metaphyseal region of the proximal femur heads of non-lame birds with attached articular cartilage caps and without visible FHN lesions on the bone surface. Cross-sectioning of the femur heads revealed that these areas lacked proper structure and contained potential necrosis, similar to the THN scores of 1 and 2 (Wideman et al., 2012) when tibias are cross-sectioned. Further investigations found more of these regions detectable via cross-sectioning in femur heads with and without articular cartilage cap separation and those with and without severe lesions on the surface of the growth plate. Therefore, we sought to document our current findings regarding the presence of internal femur head degradation and discuss the potential implications for the pathophysiology and characterization of FHN.

## 2 Materials and methods

### 2.1 Animal use and care

This study was conducted in accordance with the National Institutes of Health’s recommendations for laboratory animal use and care. All the procedures in this study were approved by the University of Arkansas Animal Care and Use Committee under protocol number 21050. Cobb 500 broilers were reared at the University of Arkansas Poultry Research farm under standard conditions. At 35 days of age, the birds (n = 24) were humanely euthanized via cervical dislocation, and the legs were excised without compromising the femur and scored for FHN, as described below. During sampling, whole legs were held on ice and then stored at −20°C until DXA analysis.

### 2.2 Bone scoring

**FIGURE 1:**
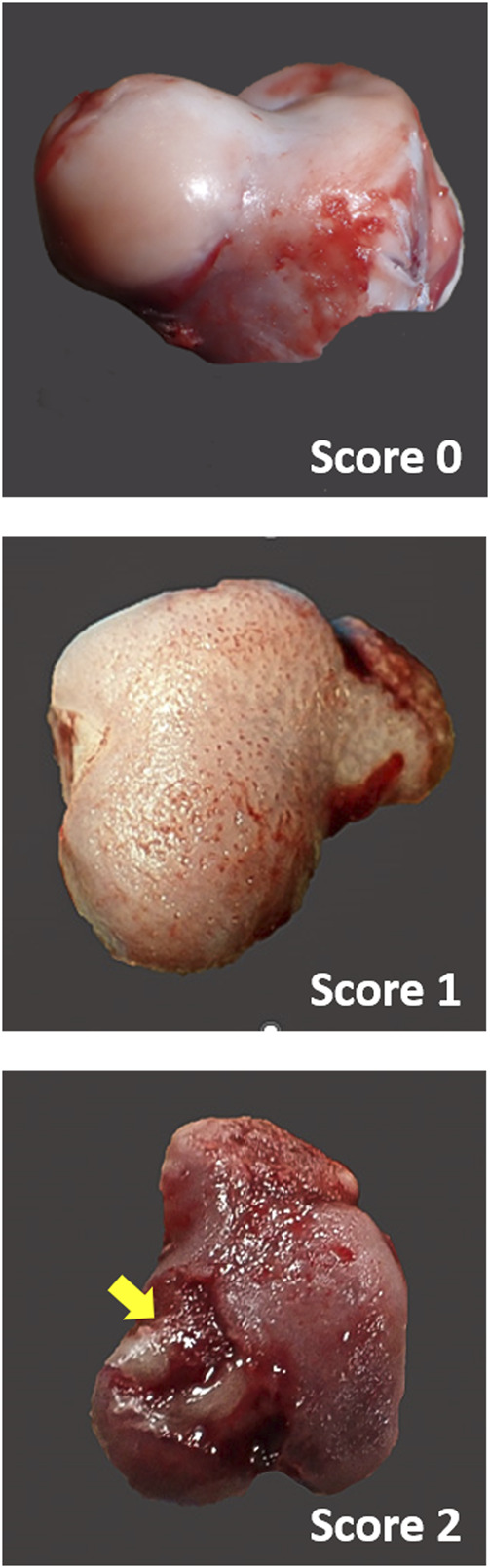
Scoring of broiler FHN. The severity of BCO lesions was assessed by a scoring scheme on a scale of 0–2. Score 0: normal bone with no abnormalities of the proximal femoral head. Bone exhibiting only articular cartilage cap separation was given a score of 1, while bone exhibiting both articular cartilage cap separation and necrotic lesions (yellow arrow) less than the size of a pencil eraser was given a score of 2.

Femoral heads were scored macroscopically by the same trained individual based on a previously reported scale (Wideman et al., 2012). In brief, normal bone, free from articular cartilage cap separation and any necrotic lesions, was given a score of 0. Bone exhibiting only articular cartilage cap separation was given a score of 1, while bone showing both articular cartilage cap separation and necrotic lesions less than the size of a pencil eraser was given a score of 2 (Figure 1).

### 2.3 DXA analysis and X-ray analysis

**FIGURE 2:**
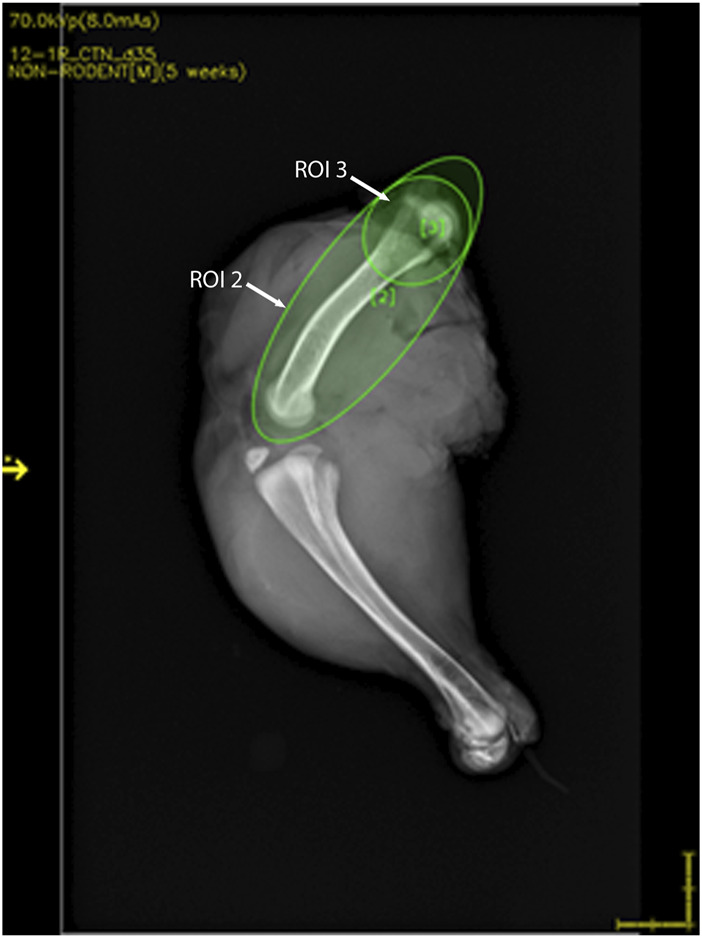
DXA image showing the representative region of interest (ROI) locations. ROI 2 includes the whole femur, whereas ROI 3 includes the femoral head.

**FIGURE 3:**
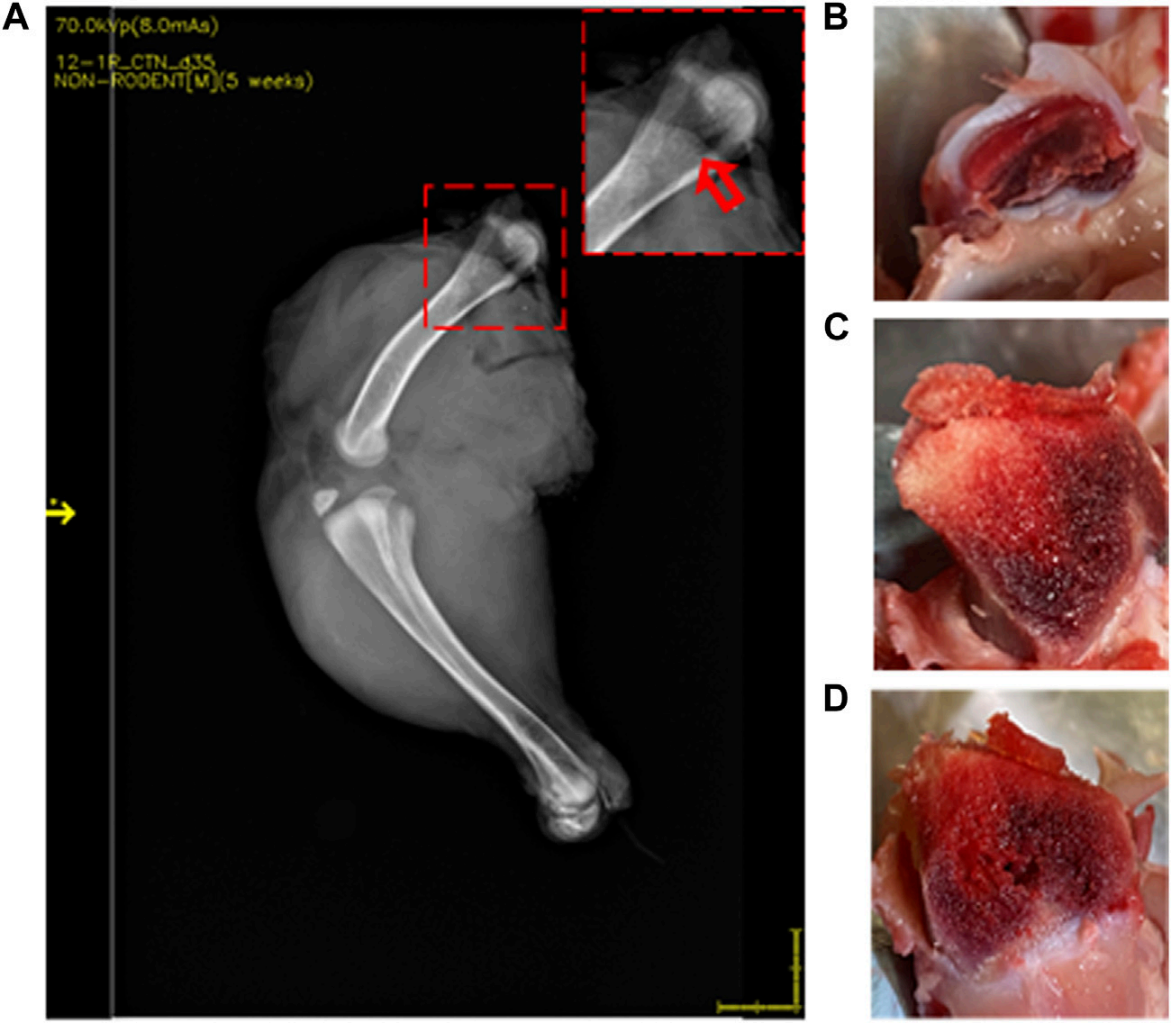
DXA images of 35-day-old broiler leg quarters with hypersignal density showing no damage in bone. Area of hypersignal density [**(A)** red arrow] and sectioning through the area in the inset with macroscopically intact bone structure **(B–D)**.

**FIGURE 4:**
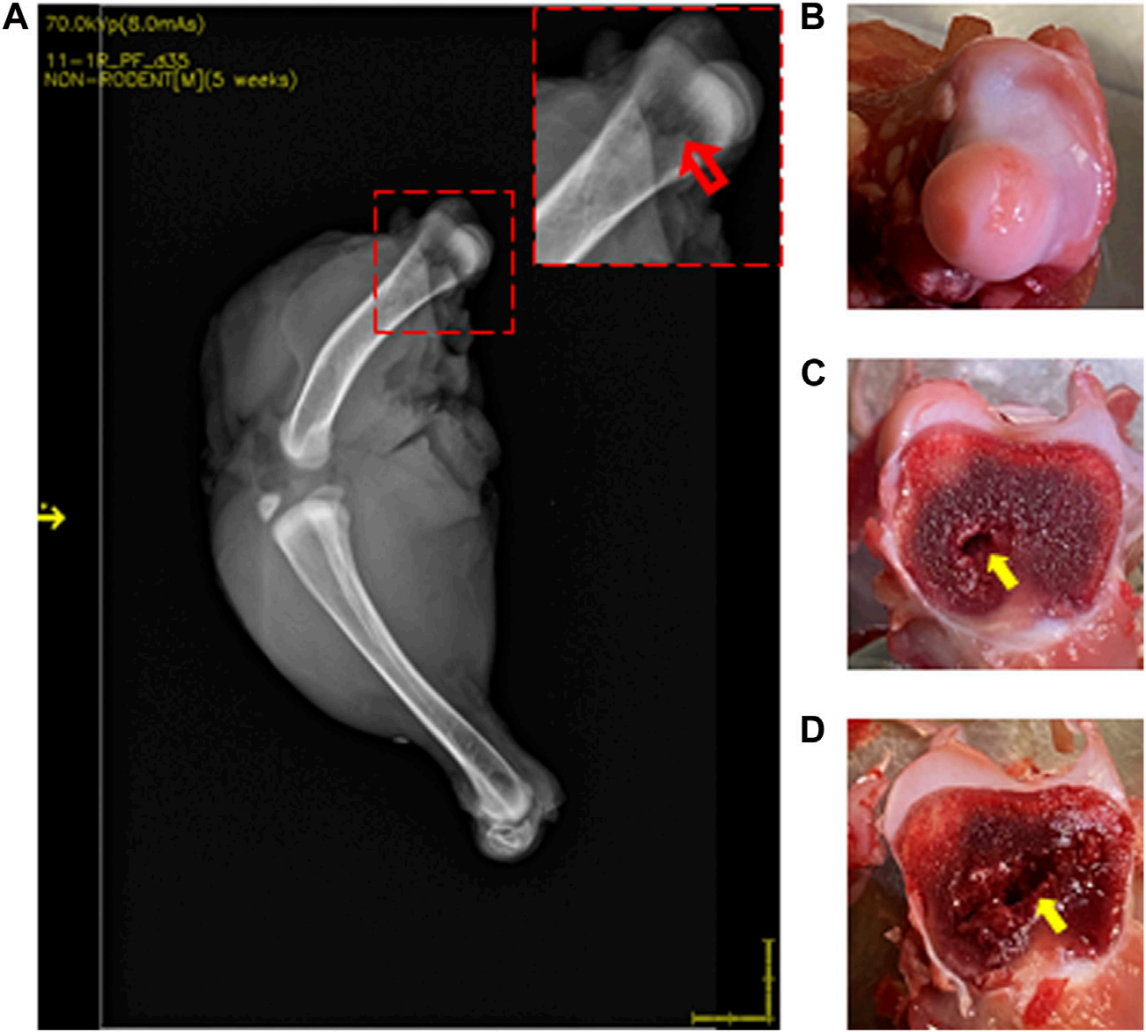
DXA images of 35-day-old broiler leg quarters with hyposignal density showing bone damage. Area of hyposignal density [**(A)** red arrow]. Intact cartilage cap, given a FHN score of 0 **(B)**. Sectioning through the area in the inset, showing visible necrosis [**(C, D)** yellow arrows].

Chicken legs were thawed overnight at 4°C. Bone mineral analyses and 2D X-ray imaging were performed using a high-resolution DXA cabinet body composition analyzer (iNSiGHT DXA, Scintica, London, ON, Canada), with the region of interest for analysis being set to include the whole femur as well as just the proximal head (Figure 2). The DXA system utilizes a cone beam X-ray, which passes through the sample, and the attenuation of the X-ray is recorded using the detector below the sample. This process provides the body composition in the x, y, and z planes and produces a 2D X-ray image (Figures 3 and 4). The acquisition parameters were as follows: 5 s at 60 kV and 5 s at 80 kV, both at 0.8 mA, with a pixel resolution of 2,048 × 2,560. There were no filters applied.

### 2.4 Statistical analysis

Femurs were categorized based on the presence (*n* = 22) or absence (*n* = 15) of a hyposignal density in the femoral head of legs with an FHN score of 0 via DXA imaging. All data are presented as the means ± SEM. The groups were compared by Student’s t*-*test using JMP Pro 17 (JMP Statistical Discovery LLC, Cary, NC), with the significance set at α = 0.05.

## 3 Results

### 3.1 DXA imaging detects femoral head lesions in 35-day-old broilers

Lesions were visualized in 22 of 37 (59.5%) femurs that were scored as normal by standard FHN scoring. Compared to normal bone (Figure 3), those with lesions showed an area of hyposignal density distal to the articular cap (Figure 4A, inset). Upon sectioning, the normal bone appeared macroscopically intact (Figures 3B–D), whereas the femurs with an area of hyposignal density showed areas of necrosis (Figures 4B–D).

### 3.2 Bone mineral content and bone mineral density of 35-day-old femurs via DXA

Bone mineral content (BMC) and bone mineral density (BMD) were measured for femurs that were scored normal (score 0) for FHN. These were then grouped into two categories: no lesion present in the DXA image or lesion evident. BMC (normalized to weight) for the whole femur and BMD of either the whole femur or just the femoral head did not differ between the legs with and without lesions. However, the femoral head of legs with an area of hyposignal density present in DXA imaging had significantly greater BMC than those without a lesion (*p* = 0.038, Table 1).

**TABLE 1:** Values for BMC normalized to weight and BMD in the three ROI regions.

	No lesion	Lesion	*p*-value
BMC (whole femur)	3.05 ± 0.10	2.85 ± 0.08	0.147
BMC (femoral head)	6.26 ± 0.25	6.95 ± 0.20	0.038
BMD (whole femur)	0.210 ± 0.005	0.208 ± 0.004	0.777
BMD (femoral head)	0.208 ± 0.006	0.206 ± 0.005	0.853

## 4 Discussion

The impressive advances achieved in the growth and productivity of the poultry industry are somewhat offset by impairments that have been acquired in other aspects of health. In particular, lameness and leg health are not only significant economic burdens to the poultry industry but also a welfare concern. As technology advances, the food-animal industry must focus on adopting new methods for identifying and characterizing both deleterious and advantageous traits. Through the use of DXA imaging, we found evidence of areas of hyposignal density in the metaphyseal region of proximal femur heads that had received a score of 0 for FHN via both 2D X-ray images collected and 3D sample composition analysis. Upon sectioning, these regions were found to be void of the proper bone structure necessary for optimal longitudinal bone growth. The region in which these lesions occurred ought to have contained a columnar array of chondrocyte cells and collagen matrix. This structure acts as the scaffolding for cortical bone laid down in the diaphysis region by the osteoblast cells (Dacke, 1998; Wideman, 2016). Without the structural support provided by the metaphyseal trabecular bone and with the disruption of the vasculature, these regions will likely progress to terminal BCO.

The proximal tibia is the hallmark for describing and observing the negative sub-growth plate effects of mechanical stress, the generation of osteochondrotic clefts or microfractures between and within the cartilage layers, and eventual necrosis as seen with advanced BCO (Farquharson and Jefferies, 2000). However, as we see here, it is likely that this also occurs within the femoral head, even beneath a clinically normal articular cap. All the legs scored 1 or 2 for FHN also showed evidence of lucent lesions, further validating DXA accuracy and use in detecting bone attrition. DXA use has already been validated in research settings for whole-body measures such as fat mass, lean tissue mass, BMC, and total body mass in poultry (Swennen et al., 2004; Schallier et al., 2019). However, this is the first report, to the best of our knowledge, to examine specifically the femoral head region of the bone using this technology. Imaging approaches used in human medicine, such as standard radiography and MRI, are unlikely to be routinely used in poultry species, even in a research setting, due to the cost of analysis as well as training and safety procedures for personnel along with optimization and calibration (Scollan et al., 1998). The equipment alone for MRI can cost up to $1,000,000, with yearly maintenance costs of over $50,000. The DXA system used here is small (16.5 cm × 25.5 cm of imaging area), with a scan time of less than 30 s, and could potentially be housed and used on-farm, with equipment and maintenance costs of around 5% of those of MRI.

There is a need for a non-terminal, non-invasive means for the genetic selection of a breeding population on leg issues, as current methods are terminal and rely on using siblings. Though here we used excised legs, the DXA system has the potential to be used in live birds as well. A compact unit can fit and image a young bird, allowing for its possible use in identifying problematic birds at an earlier age and removing them from breeding cohorts. There is evidence of bone necrosis and BCO in chicks as early as 14 days post-hatch at necropsy (Wideman, 2016). Additionally, as leg problems in young poultry are typically associated with the proximal, weight-bearing ends of the femur, tibia, or tibiotarsus (Packialakshmi et al., 2015; Wideman, 2016), this technology allows for the visualization of all of these areas simultaneously for more efficient screening as necessary. The use of this technology can help in understanding the progression of FHN/BCO, particularly in understanding the circulating factors such as cytokines and chemokines that, to date, have only been able to be assessed at terminal stages (Ramser et al., 2021). Additionally, although we focused on bone parameters, the DXA system also obtains fat and lean mass, which may have further applications in poultry.

Interestingly, the BMC, as normalized to the weight of the femoral head in bones with internal lesions, was greater than that of those that appeared normal via the DXA scan. In a study of sickle cell disease-induced avascular necrosis of the femoral head in humans, the carbonate to phosphate ratio favored carbonate, which indicates differences in crystallinity and a more brittle bone matrix (Al-Ghaithi et al., 2021), which may impact the changes in BMC. Further investigation into the underlying cause of these BMC differences is warranted, as it may relate to the underlying structural differences that increase susceptibility to FHN or may be a consequence of lesion presence. Surprisingly, no differences were seen in BMD, a measure often associated with structure and mineralization (Boskey et al., 1999). Similar to the results here, no differences were seen in the BMD of the necrotic lesion of patients with pre-collapse osteonecrosis of the femoral head compared to that of healthy controls (Baba et al., 2020), suggesting that disruptions to the microarchitecture of bone collagen structure may be masked by this measure.

In conclusion, we have shown a novel use of DXA technology for the study of bone attrition in poultry and identified previously overlooked sub-clinical lesions within the femoral head of growing broilers. Further research may allow for the study of the onset and progression of FHN/BCO and hold the potential for aiding in selection programs.

## Data Availability

The original contributions presented in the study are included in the article/Supplementary material; further inquiries can be directed to the corresponding author.
